# Transforming Growth Factor-β2 protects the small intestine during methotrexate treatment in rats possibly by reducing stem cell cycling

**DOI:** 10.1038/sj.bjc.6600342

**Published:** 2002-07-15

**Authors:** B van't Land, H P Meijer, J Frerichs, M Koetsier, D Jager, R L Smeets, L M'Rabet, M Hoijer

**Affiliations:** Numico-Research, Department of Condition and Disease Specific Research, Bosrandweg 20, PO Box 6700 CA Wageningen, The Netherlands

**Keywords:** Transforming Growth Factor β2, oral supplementation, chemotherapy-chemoprotection, G1-phase growth arrest

## Abstract

During chemo- and radiation therapy, the balance between epithelial cell proliferation, differentiation, and cell death at the villus tip is disrupted by premature death of dividing epithelial cells. This will subsequently lead to the onset of mucosal barrier injury in the whole gastrointestinal tract. Up till now there is no validated method to treat side effects occurring due to therapy. An approach to manage this side effect might be to reversibly arrest growth of epithelial stem cells during therapy using Transforming Growth Factor-β2. A Transforming Growth Factor-β2 enriched fraction prepared from bovine milk was shown to protect small intestinal epithelial cells against cell cycle specific chemotherapeutic agents by arresting the cells in G1-phase. Secondly, in a rat model for induced small intestinal damage, oral supplementation of rats exposed to methotrexate with the Transforming Growth Factor-β2 enriched fraction significantly reduced the chemotherapy-associated weight loss and ileal villus atrophy by reducing cell proliferation in the normal stem cell population. Thus oral supplementation with a bovine milk fraction enriched for Transforming Growth Factor-β2 attenuated the side effects of chemotherapy in the small intestine in rats.

*British Journal of Cancer* (2002) **87**, 113–118. doi:10.1038/sj.bjc.6600342 www.bjcancer.com

© 2002 Cancer Research UK

## 

Anti-cancer chemotherapy and radiation treatment relies primarily on the high proliferation rate of tumour cells. Stem cells of the gastrointestinal tract are among the most rapidly proliferating cells in the body, and therefore also affected by this anti-tumour approach targeted towards proliferating cells. Entrocyte function in rats has been shown to be effected adversely by methotrexate (MTX) resulting in altered intestinal permeability (Taminiau *et al*, 1980) which has a counterpart in humans (Keefe *et al*, 1997; Parrilli *et al*, 1989). The several complications, i.e. vomiting, nausea and mucosal barrier injury (Sonis, 1998; Blijlevens *et al*, 2000) result from loss of integrity of the intestinal barrier which is a key element in preventing inappropriate uptake and transport of macromolecules, bacteria and enteric toxins. To date, there is still no validated method to treat the side effects of anti-cancer treatment.

Specific cell cycle arrest of rapid proliferating epithelial stem cells is a promising approach to protect the mucosa from the direct side effects of anti-cancer treatment. Transforming Growth Factor-β3 (TGF-β3) has been shown to prolong or arrest the cell cycle of epithelial cells in the G1-phase thereby providing protection *in vitro* against cell-cycle specific chemotherapeutic agents that act predominantly in the S- or M-phase of the cell cycle (McCormack *et al*, 1997). Topical administration of recombinant human TGF-β3 has also been shown to attenuate the oral mucositis induced in hamsters by 5-Fluorouracil with a concomitant reduction in weight loss and shorter duration of oral mucositis (Sonis *et al*, 1997; Spijkervet and Sonis, 1998). It has been suggested that this protection is mediated by the inhibition of proliferation of basal epithelial cells during exposure to chemotherapy. Positive results have been obtained in humans using mouthwashes containing recombinant TGF-β3 (Wymenga *et al*, 1999), indicating a promising treatment against the oral complications of anti-cancer therapy. Administration of recombinant TGF-β3 directly into the small intestine of mice had a protective effect during radiation (Potten *et al*, 1997) by reducing stem cell cycling (Booth *et al*, 2000). To our knowledge however, no study has been done investigating the potential protective effects of orally administrated TGF-β2 originating from a natural source. We therefore set out to determine the efficacy *in vitro* and *in vivo* of TGF-β2 in protecting intestinal epithelial cells from damage induced by methotrexate (MTX).

## MATERIALS AND METHODS

### Cells

The small intestine rat epithelial cell line (IEC-6) was obtained from ATCC (Rockville, MD, USA) and cultured at 37°C and 5% CO_2_ in Dulbecco's modified Eagle's medium adjusted to contain 1.5 g l^−1^ sodium bicarbonate and 4.5 g l^−1^ glucose and supplemented with 50 000 IU l^−1^ penicillin, 50 mg l^−1^ streptomycin and 10% heat inactivated foetal calf serum (FCS) all obtained from Gibco BRL. Cells were used between passage 19 and 35.

### TGF-β2 enriched fraction

TGF-β2 enriched fraction was obtained from bovine milk by purification with cationic exchange chromatography (DMV international). The TGF-β2 enriched fraction contained 750 μg g^−1^ TGF-β protein consisting for ∼90% TGF-β2 and ∼10% TGF-β1. No detectable levels of TGF-β3 could be measured by ELISA techniques. Forty-nine per cent of the TGF-β in the fraction was unbound and active as measured in a bioassay.

### Proliferation assay

IEC-6 cells were cultured for 72 h in a 96-well microtiter plate (Costar, Schiphol, The Netherlands) with a concentration range of 0.25–2.5 ng ml^−1^ of recombinant TGF-β3 or TGF-β2 in the enriched fraction. Sixteen hours before the end of the assay 10 μM 5-bromo-2′-deoxyuridine (BrdU) was added to the wells, and the incorporation was visualised according to the manufacture protocol (Boehringer Mannheim), and measured at 450 nm.

### Cell death/cell cycle arrest

To establish the capacity of TGF-β2 enriched fraction to arrest cells in growth, flowcytometry analyses were done. IEC-6 cells were cultured in a 6 well plate and incubated together with 2.5 μg ml^−1^ cytosine arabinoside (Ara-C, cytarabine; Sigma, Zwijndrecht, The Netherlands) for 48 h with or without TGF-β2 enriched fraction (2 ng ml^−1^ of TGF-β2) or 2 ng ml^−1^ recombinant TGF-β3. The cells were then harvested and prepared for flowcytometry as described previously (Fraker *et al*, 1995). Briefly, cells were trypsinised, washed twice with PBS containing 0.1% EDTA (PBS-E) then fixed in 70% ethanol for 45 min at −20°C. Cellular DNA was labelled with propidium iodide by washing the cells twice with PBS-E and incubating them with PBS-E containing propidium iodide (5 μg ml^−1^) and RNA-se (1 μg ml^−1^) for 45 min in the dark. The cell preparations were analysed using a flowcytometer (Coulter, Mijdrecht, The Netherlands) and were separated into three populations according to their fluorescence intensity i.e. DNA content N=1 represent cells in the G1-phase, N=2 mitotic G2-phase and apoptotic cells contain N<1 amount of DNA.

### **In vitro** cell viability assay

IEC-6 cells were harvested in the exponential phase of growth, deposited in 96-well microtitration plates (Costar, Schiphol, The Netherlands) and cultured for 24 h with or without TGF-β2 enriched fraction (2 ng ml^−1^ TGF-β2). A concentration range of 0.05–0.13 μg ml^−1^ Cytosine Arabinoside (Ara-C) (Sigma, Zwijndrecht, The Netherlands) was subsequently added to the cells and incubated for 72 h at 37°C and 5% CO_2_. The cells were then washed three times with PBS and incubated in normal medium for 48 h at 37°C and 5% CO_2_. After 4 h incubation at 37°C with 50 μl tetrazolium salt 2,3-bis[2-methoxy-4-nitro-5-[(sulphenylamino)carbonyl]-2H-tetrazolium-hydroxide] (XTT) solution as provided in the kit from Boehringer Mannheim, formation of the formazan dye by metabolically active cells was measured at 490 nm. To establish that TGF-β2 is the active compound in the fraction, neutralising monoclonal mouse anti-TGF-β2 (Genzyme Diagnostics, Leuven, Belgium) was added at a concentration of 0.1 mg ml^−1^ for 1 h, prior to testing in the cellular viability assay as described above.

### **In vitro** protease degradation assay

*In vivo* protein degradation in the stomach was simulated by incubation of the TGF-β2 fraction with a buffer containing 53 mmol l^−1^ NaCl, 0.7 mmol l^−1^ CaCl_2_, 15 mmol l^−1^ KCl, 71 mmol l^−1^ NaHCO_3_, 70 μg ml^−1^ pepsin from bovine pancreas (Sigma, Zwijndrecht, The Netherlands) and 70 μg ml^−1^ lipase (Sigma, Zwijndrecht, The Netherlands) at pH 3.0 for 60 min at 37°C. After incubation, the enzymes were inactivated by neutralising with NaOH to pH 7.0, and stored at −20°C until required for analysis. The remaining cytoprotective capacity of the fractions after protein degradation was tested in the cell viability assay as described before.

### MTX-animal model

The animal experiments were approved by the Animal Care and Ethics Committee (DEC, The Netherlands) and were performed according to the UKCCCR ‘Guidelines for the Welfare of Animals in Experimental Neoplasia’ (1998). Eight-week old female Wag/Rij rats (Charles River, Wiga, Germany) with a weight average of 119.0±4.5 g were kept in pairs in conventional cages and in a 12 h reversed light/dark cycle, with free access to chow and water. All rats switched to a semi-synthetic diet (Hope-farms Woerden, The Netherlands) containing 1 mg kg^−1^ folic acid 7 days before starting the experiments. At the same time they were trained to expect to be given a supplement twice daily at 0900 h and 1600 h using a placebo containing 2.5% casein, 2.5% sucrose and strawberry vanilla flavour dissolved in water, which was added in addition to normal chow. Placebo and semi-synthetic diet were given to the animals until the end of the experiment. Supplementation of TGF-β2 enriched fraction (5 μg TGF-β2/rat/day) was started on day −1 until day 1. Two groups of rats (*n*=4 per group) supplemented with placebo or the TGF-β2 enriched fraction were injected i.v. on day 0 with 20 mg kg^−1^ body weight MTX (Ledertrexate SP Forte, AHP Pharma Hoofddorp, The Netherlands), followed by a second MTX injection of 10 mg kg^−1^ body weight 24 h later. Two groups of control animals (*n*=4 per group) were injected with a similar volume of 7% saline solution and supplemented with either placebo or the TGF-β2 enriched fraction. Body weight was monitored daily. In a pilot experiment (data not shown) it was found that maximal intestinal damage occurred on day 4, thus small intestine of each animal was collected subsequently at this time thereafter. Rats were injected with 50 mg kg^−1^ body weight BrdU i.p. 16 h before tissue collection to determine the effects on cellular proliferation and to establish the effect of the TGF-β2 enriched fraction on intestinal epithelial cell proliferation two additional groups (*n*=4) of animals were scarified 16 h after the last supplementation with the TGF-β2 enriched fraction. Animals were killed under sedation and segments of the small intestine were removed for immunohistological staining and morphological examination. All segments were carefully rinsed once with cold PBS and fixed in 4% paraformaldehyde at pH 7.4 (Sigma, Zwijndrecht, The Netherlands) for 16 h, dehydrated and embedded in paraffin.

### Immunohistology

To examine morphological damage in the small intestine, 5 μm sections were cut transversely to visualise the entire villus, subsequently rehydrated and stained with 1 mg ml^−1^ Haematoxylin (Sigma, Zwijndrecht, The Netherlands) and 10 mg ml^−1^ Eosin (Sigma, Zwijndrecht, The Netherlands). BrdU incorporation was detected using 0.5 μg ml^−1^ mouse monoclonal anti-BrdU (Boehringer Mannheim) followed by incubation with 1.5 μg ml^−1^ anti-mouse IgG (Vector Laboratories), which was visualised after thorough washings with PBS using ABC-complex (Vector Laboratories) and 3,3′-diaminobenzidine tablets dissolved in H_2_O (Sigma, Zwijndrecht, The Netherlands). Villus lengths, crypt depths and BrdU-incorporation were measured with an image analysis program (Zeiss KS300). At leased 15 cross-sections per rat were analysed blindly by two independent researchers.

### Statistical analysis

The Student two sided *t*-test was used to analyse cellular viability (expressed as a per cent of control) between cell cultures treated with chemotherapeutic agents and untreated control cultures, villus height and crypt depth measurements (mm) and body weight (expressed as per cent of day 0 control) between the groups of animals supplemented with TGF-β2 enriched fraction and those that were not. *P* values<0.05 were considered statistically significant.

## RESULTS

### The proliferation inhibition capacity of the TGF-β2 enriched fraction

**Figure 1:**
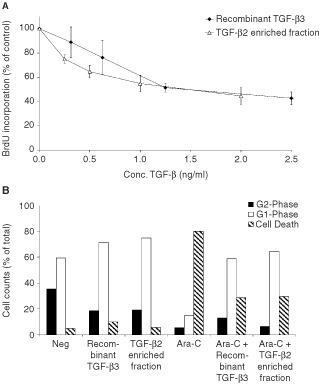
(**A**,**B**) TGF-β2 enriched fraction and recombinant TGF-β3 comparably inhibit proliferation and protect cells against Ara-C induced toxicity by induction of G1-phase arrest. In Figure 1A, BrdU incorporation into IEC-6 cells incubated for 48 h with a concentration range of recombinant TGF-β3 (closed triangles) or the TGF-β2 enriched fraction (open triangles) is shown. The data represent mean±s.d. of three independent experiments. In Figure 1B, G1-phase arrest is detected by flowcytometry-analysis of propidium iodide labelled IEC-6 cells incubated with recombinant TGF-β3 or the TGF-β2 enriched fraction in the presence or absence of Ara-C. A total of 10 000 cells were counted and divided into three sections, of which G1-phase represents cells with N amount of DNA, G2-phase represents the cells in proliferating phase i.e. containing N2 amount of DNA and the remaining dying cells (amount <N).

A dose dependent proliferation inhibition up to 56±5% was seen when IEC-6 cells were incubated with 2.0 ng ml^−1^ TGF-β2 in the enriched fraction. The *in vitro* proliferation inhibition capacity of the TGF-β2 enriched fraction is comparable to equal concentrations of recombinant TGF-β3 (Figure 1A

). When IEC-6 cells were incubated with recombinant TGF-β3 a decrease of 17.2% of the proliferating cells was seen in the flowcytometry analysis (Figure 1B), which resulted in an increase in the G1-phase population. A comparable shift of 16.4% from proliferating cells to the G1-phase was seen with the TGF-β2 enriched fraction. These results indicated that proliferation of the IEC-6 cells is inhibited by the TGF-β2 enriched fraction, due to growth arrest in the G1-phase.

### Protective effect of TGF-β2 enriched fraction on cell cycle-specific drug-induced damage

**Figure 2:**
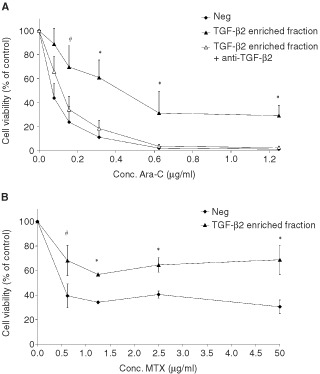
(**A**,**B**) Epithelial cells are protected against cell cycle specific drug, due to TGF-β2 present in TGF-β2 enriched fraction. Cell viability of IEC-6 cells after treatment with a concentration range of Ara-C (A) or MTX (B), incubated with (closed triangle) or without (closed square) TGF-β2 enriched fraction is shown. The open triangles (A) show cells, which were incubated with TGF-β2 enriched fraction after blocking of TGF-β2 activity by pre-incubation with anti-TGF-β2 antibodies. Mean±s.d. of three individual experiments is shown. Statistical differences between viability of cells incubated with TGF-β2 enriched fraction after depletion for TGF-β2 and control or TGF-β2 alone are indicated with #(*P*<0.05) or *(*P*<0.01).

An Ara-C dependent increase was seen in the fraction of apopotic cells on flowcytometry (Figure 1B) and a concentration dependent decline was seen in the cellular viability measured using XTT reagent (Figure 2A

). In both assays inhibition of the toxic effect was seen when cells were incubated with TGF-β2 enriched fraction in comparable efficiency as recombinant TGF-β3. The IC-50 for with Ara-C is 0.07 μg ml^−1^ in the absence of TGF-β2 enriched fraction, whereas treatment in presence of the TGF-β2 enriched fraction gave a six-fold higher IC-50 value (0.43 μg ml^−1^) indicating a higher cell survival. Even with high concentrations of Ara-C (2.5 μg ml^−1^), 30% of the cells remained viable when simultaneously incubated with the TGF-β2 enriched fraction. Incubation with TGF-β2 enriched fraction also showed a diminished reduction in cell viability of 28±7% when IEC-6 cells were incubated with MTX, yet another cell cycle specific drug (Figure 2B).

The bovine milk fraction although enriched for TGF-β2, might still contain other active proteins. The cytoprotective effect of the TGF-β2 enriched fraction was completely blocked by pre-incubation of the fraction with a specific monoclonal anti-TGF-β2 antibody. There was as little as 1% difference seen between the cells incubated with TGF-β2 enriched fraction which was neutralised for TGF-β2 and the cells treated with high doses of Ara-C alone (see Figure 2A).

**Figure 3:**
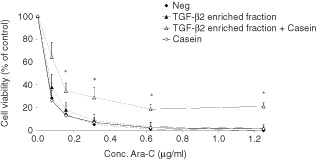
Diminished degradation of TGF-β2 due to the presence of casein. Cell viability of IEC-6 cells after treatment with a concentration range of Ara-C, of which the triangles represent treatment with TGF-β2 enriched fraction in an *in vitro* degradation assay, with (open) or without (closed) casein present during the degradation. The controls with (open), or without (closed) casein during the *in vitro* degradation assay, are represented by squares. Mean±s.d. of three separate experiments are shown. *Indicates significant differences with the negative control group (*P*<0.01).

Since the use of TGF-β2 enriched fraction in enteral nutrition was examined, in an *in vitro* protease degradation (as described in Materials and Methods), the effect of intestinal transit and degradation was evaluated. The remaining protective capacity of TGF-β2 enriched fraction against Ara-C induced toxicity was tested on the IEC-6 cells after *in vitro* degradation with or without the presence of casein. Without the presence of casein, the protective effect of TGF-β2 enriched fraction was indeed decreased after *in vitro* degradation to 3–4% and was totally diminished at higher concentrations of Ara-C (Figure 3

). Within the presence of casein however, the protective effect of TGF-β2 enriched fraction remained intact after the *in vitro* degradation, as it resulted in 21±3% cell viability left after exposure to the high concentrations of Ara-C.

### Protective effects of TGF-β2 enriched fraction **in vivo**

**Figure 4:**
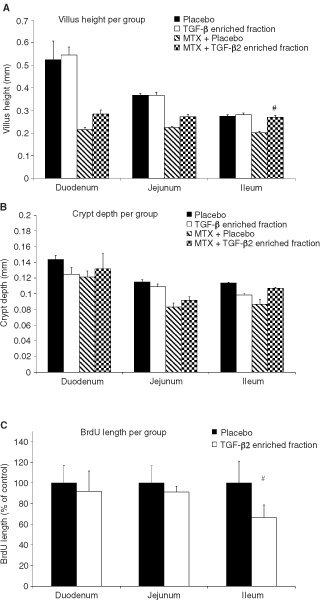
(**A**,**B**,**C**) Supplementation of TGF-β2 enriched fraction reduced MTX induced villus atrophy due to stem cell proliferation inhibition. Villus length (**A**) and crypt depths (**B**) were measured from different regions in the small intestine at day 4 (*n*=4 per group) from rats receiving either placebo (closed bars) or the TGF-β2 enriched fraction (open bars). Statistical differences between the MTX control group only receiving placebo (hatched bars) or TGF-β2 enriched fraction (squared bars) are indicated with #(*P*<0.05). At least 60 microscopical measurements were made per animal, at a magnification of 10× per rat. Bars show mean±s.d. for three rats per group. In Figure (**C**), BrdU incorporation is shown as length in per cent of control, measuring from the lowest BrdU positive crypt cell until the highest BrdU positive cell upon the villus axis. Lengths were measured in different regions of the small intestine at day two from four rats per group receiving placebo (closed bars) or TGF-β2 enriched fraction (open bars). Statistical differences are indicated with #(*P*<0.05).

Throughout the entire small intestine MTX caused a shortening of villus length, to a minimum of 0.2 mm was seen due to MTX toxicity (Figure 4A

). In the duodenum the villus length was reduced for >50%, in the jejunum for ∼40% and in the ileum a reduction of ∼25% was detected. Supplementation with TGF-β2 enriched fraction resulted in restoration of villus length throughout the small intestine, and reached significance (*P*<0.05) in the ileum. Villus length was reduced to 0.20±0.010 mm in the MTX treated group and restored to 0.27±0.009 mm in the rats supplemented with TGF-β2 enriched fraction, which equals the villus length in the control rats (0.27±0.006 mm). However no significant restoration of villus atrophy seen after MTX treatment could be detected in the duodenum or jejunum. Equal protective capacities of the TGF-β2 enriched fraction were found in a second set of experiments (data not shown). There was no effect seen on villus length in control rats supplemented with TGF-β2 enriched fraction (Figure 4A). The effect of MTX on the crypt depths is shown in Figure 4B. No significant effect of the TGF-β2 enriched fraction supplementation could be detected in the crypt depth measurements. The proliferation inhibition capacity of TGF-β2 enriched fraction *in vivo* was estimated using BrdU incorporation during the 16 h after the last supplementation. The BrdU length was measured starting from the lowest BrdU positive crypt cell until the highest BrdU positive cell migrated upon the villus axes. As can been seen in Figure 4C a significant (*P*<0.05) inhibition of proliferation could be detected in the ileum of rats supplemented with the TGF-β2 enriched fraction compared to the control group.

**Figure 5:**
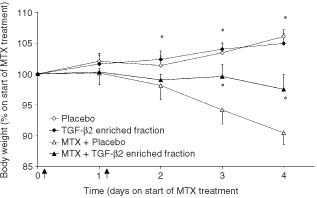
Weight loss due to MTX toxicity is reduced by supplementation of TGF-β2 enriched fraction. Effect of MTX on the weight of rats is shown as per cent of weight on day 0 (mean±s.d.). The arrows located on the X-axis indicate the two MTX injections. Two groups of rats treated with or without MTX are shown in triangles and squares respectively, in which the closed symbols represent groups supplemented with TGF-β2 enriched fraction. *Indicates significant differences between the MTX treated group receiving placebo and the other groups. (*P*<0.01).

All the control animals not receiving MTX, showed a steady growth, which was unaffected by the supplementation of TGF-β2 enriched fraction (Figure 5

). The rats however treated with MTX showed a reduction in body weight already after the first MTX injection, resulting in difference of 15±2% with the control rats at day 4. Supplementation with TGF-β2 enriched fraction during MTX-treatment showed significantly less reduction in body weight than animals receiving placebo. The average body weight loss of the supplemented rats was only ∼3%, whereas rats receiving only MTX lost ∼10% of their body weight.

## DISCUSSION

The small intestine maintains its function due to solid regulation between epithelial cell proliferation, differentiation, and cell death at the tip of the villus. During cytostatic chemotherapy, this balance is disrupted by early cell death of the dividing stem cells located in the crypt area. The present study shows that reducing epithelial stem cell proliferation with TGF-β2 enriched milk fraction from bovine origin protects the small intestinal epithelial cell *in vitro* as well as *in vivo* from this premature death. It was shown that the proliferation inhibition and cytoprotection of the TGF-β2 enriched fraction is as effective as recombinant TGF-β3. Furthermore immuno-neutralisation of TGF-β2 from the enriched fraction proved that it is the TGF-β2 in the fraction that protects the cells from cell-cycle specific drug-induced cell death, i.e. Ara-C (Figure 2A) and MTX (Figure 2B) *in vitro*.

The protective mechanisms of growth factors used in animal models of mucosal barrier injury are likely to be multifactoral. The benefit of pre-treatment with IL-11 and KGF has been shown to modulate chemotherapy- or radiation-induced intestinal epithelial injury (Potten, 1995, 1996; Farrell *et al*, 1998). The mechanism whereby by IL-11 protects remains to be elucidated, but the protection mediated by TGF-β3 and KGF appears to be due to a reduction in stem cell proliferation (Booth *et al*, 2000; Potten *et al*, 2001). It is known that TGF-β3 reversibly arrests cell growth in the G1-phase (Ko *et al*, 1998; Warner *et al*, 1999) which was confirmed for the TGF-β2 enriched fraction (Figure 1B). Reduced proliferation of stem cells *in vivo* could also be detected after supplementation of TGF-β2 enriched fraction showing limited BrdU incorporation (Figure 4C). The significant protection of TGF-β2 enriched fraction seen on villus atrophy was limited to the ileum, which was also the region where significant reduction of proliferation was found which indicates that protection is brought about by reduced stem cell proliferation. Cell cycle specific chemotherapeutic agents like Ara-C and MTX, inhibit specific enzymes, leading to inhibition of DNA replication with S phase specificity. These processes are reduced in cells with arrested growth in the G1-phase by TGF-β2. In accordance with our results epithelial cell proliferation stimulation during chemotherapy treatment with IGF-1 is not favourable (Howarth *et al*, 1998). IGF-1 given after chemotherapy however resulted in an improved regeneration of small intestinal damage (Taylor *et al*, 2001). A study done by Howarth *et al* (1996) using a growth factor enriched milk fraction, which offered a slight protection against MTX induced damage *in vivo*. TGF-β2 mediated G1-phase cell-cycle arrest could not explain this protective effect since the fraction in question actually stimulated the growth of epithelial cells *in vitro* rather than arresting it.

The length of the villus of the duodenum is twice as long as that of the ileum which might reflect a higher proliferating rate of the epithelial cells, or the presence of more crypts containing stem cells per villi in the duodenum than in the ileum. Equal damage to the epithelial cells after exposure to MTX might explain why villus length was reduced two-fold in the duodenum compared to the ileum 4 days after MTX treatment. Strangely, however, unlike what was seen in the ileum, villus length in the duodenum did not return to its normal length after supplementation with the TGF-β2 enriched fraction. Differences in TGF-β2 induced proliferation inhibition efficiency might be related to binding with casein, rendering it less free and therefore less active at the beginning of the small intestine. This difference in villus restoration could also reflect diversity in TGF-β receptor expression throughout the small intestine. TGF-β3 and TGF-β1 show preferential binding to the RI and RII signalling complex, whereas TGF-β2 requires RIII for presentation to the RI/RII complex in order to initiate the cascade of events leading to proliferation inhibition (Moustakas *et al*, 1993; Henis *et al*, 1994). The expression of TGF-β receptors on epithelia in the small intestine is currently under investigation. Although a significant restoration of the villus atrophy could only be detected in the ileum, the weight of animals receiving TGF-β2 enriched fraction did not show the same reduction as seen in MTX treated animals receiving placebo which indicates that supplementation of TGF-β2 enriched fraction attenuates small intestinal function loss induced by MTX.

Safety and tolerability studies are required before oral supplementation of TGF-β2 enriched fraction can be used clinically. In addition, supplementation of any product reducing side effects of anti-tumour treatment should not influence the treatment on cancer cells negatively. A phase I study has already been conducted to determine the safety and tolerability of recombinant TGF-β3 mouthwashes for prevention of chemotherapy induced oral mucositis (Wymenga *et al*, 1999). In this study neither systemic absorption nor development of TGF-β3 antibodies were observed. It should be considered however, that the mouthwashes were not ingested, which is essential for TGF-β2 which is intended for the treatment of mucosal barrier injury throughout the whole gastrointestinal tract. On the other hand, a relative low dose of TGF-β2 could be used, since systemic uptake of TGF-β2 is not required to protect the intestinal epithelial stem cells. There is a strong correlation between progressive malignancy and loss in sensitivity towards the negative regulation of cellular proliferation TGF-β in tumour cells, which is often due to mutational inactivation or decreased TGF-β receptor function (de Caestecker *et al*, 2000; Kim *et al*, 2000). In addition tumour cells often escape from the anti-proliferative effects of TGF-β by mutation or disregulated expression of components in its signalling pathway (Yanagisawa *et al*, 2000). These publications suggest that TGF-β2 could be given safely during anti-tumour therapy. In conclusion, the present results indicate that oral supplementation of bovine TGF-β2 enriched milk fraction might prove useful in ameliorating the complications of anti-cancer therapy in humans with TGF-β2 unresponsive tumour cells.
